# Impact of SARS-CoV-2 Pandemic on Bronchiolitis Hospitalizations: The Experience of an Italian Tertiary Center

**DOI:** 10.3390/children8070556

**Published:** 2021-06-28

**Authors:** Giacomo Stera, Luca Pierantoni, Riccardo Masetti, Davide Leardini, Carlotta Biagi, Danilo Buonsenso, Andrea Pession, Marcello Lanari

**Affiliations:** 1 Residency School of Pediatrics, University of Bologna, 40138 Bologna, Italy; giacomo.stera@studio.unibo.it (G.S.); davide.leardini3@studio.unibo.it (D.L.); 2 Pediatric Emergency Unit, IRCCS Azienda Ospedaliero-Universitaria di Bologna, 40138 Bologna, Italy; carlotta.biagi@aosp.bo.it (C.B.); marcello.lanari@unibo.it (M.L.); 3 Pediatric Oncology and Hematology “Lalla Seràgnoli”, Pediatric Unit, IRCCS Azienda Ospedaliero-Universitaria di Bologna, 40138 Bologna, Italy; riccardo.masetti5@unibo.it (R.M.); andrea.pession@unibo.it (A.P.); 4 Department of Woman and Child Health and Public Health, Fondazione Policlinico Universitario A. Gemelli IRCCS, 00168 Rome, Italy; danilobuonsenso@gmail.com; 5 Istituto di Microbiologia, Università Cattolica del Sacro Cuore, 00168 Roma, Italy

**Keywords:** coronavirus, COVID-19, non-pharmaceutical interventions, pediatric emergency department, infants, respiratory syncytial virus

## Abstract

SARS-CoV-2 pandemic restrictions have deeply altered the common respiratory illnesses burden. The aim of this paper was to clarify how these measures may have influenced bronchiolitis epidemiology, exploring possible explanations. We studied 342 infants hospitalized for bronchiolitis at our center from four different epidemic seasons (October–April 2017–2018, 2018–2019, 2019–2020 and 2020–2021). March–April hospitalization rate, RSV (respiratory syncytial virus) infection, pediatric intensive care unit (PICU) admission and oxygen therapy administration data were compared among different seasons to outline any changes during the SARS-CoV-2 outbreak. In March–April, 30 (23.1%), 28 (24.6%) and 5 (5.1%) infants were hospitalized for bronchiolitis, respectively, in 2017–2018, 2018–2019 and 2019–2020, with a lower rate in March–April 2020 (*p* < 0.001). No hospitalizations for bronchiolitis occurred during the epidemic season of 2020–2021. No significant differences in RSV infections, oxygen therapy administration and PICU admissions across seasons were outlined. In conclusion, we report a severe decrease in hospitalizations for bronchiolitis at our center throughout the entire SARS-CoV-2 outbreak rather than only during the lockdown periods. This seems to suggest a pivotal role for the systematic implementation of cost-effective non-pharmaceutical interventions (NPIs) such as compulsory face masks and hand hygiene, which were deployed for the entire pandemic, in reducing the circulation of infectious agents.

## 1. Introduction

Bronchiolitis is one of the most common respiratory illnesses, affecting about 20% of the annual birth cohort and leading to 2–3% of the hospitalization rate in children younger than 12 months every year. Presentation occurs in epidemics, mainly following the prevalence of the respiratory syncytial virus (RSV), with higher rates from November to March, with slight variability through the years [1]. In March 2020, the Emilia-Romagna (E-R) region, Northern Italy, had one of the largest clusters of SARS-CoV-2 in the world [2]. Official up-to-date statistics show the number of total cases and deaths related to the virus, respectively, to be 4,153,374 and 124,063 in Italy (total population: 59,257,600), and 379,130 and 13,081 in the E-R region (total population: 4,450,500) [2,3]. During the pandemic, a relevant decrease in the number of attendances to pediatric emergency departments (PEDs) was observed in Italy during March 2020 compared with the same period in 2018 and 2019 [4]. Concomitantly, we witnessed a drop in hospitalizations for bronchiolitis to our PEDs, which occurred in advance of the expected end of the epidemic season. To date, several papers have described the reduction in the burden of common diseases, including bronchiolitis, during the SARS-CoV-2 era, focusing mainly on the overlapping end of the 2019–2020 bronchiolitis season with the so-called “first wave” of SARS-CoV-2 in March 2020 [5,6]. Interestingly a strong reduction in the burden of common seasonal infections, including bronchiolitis, following the deployment of social distancing measures has been reported worldwide, despite wide variability in the population characteristics and in the severity of the restrictions considered. Data from Finland, often regarded as one of the European countries favorable to a less restrictive approach, describe an early ending of the influenza epidemic season, together with a strong reduction in the bronchiolitis burden as of March 2020 with the social distancing measures in place [7]. Similar findings have been outlined by Hatoun et al. in North America with a bronchiolitis diagnosis rate of 100,000 patients/day, decreasing from 20.1 to 0.6 after the general lockdown imposed during the first SARS-CoV-2 wave in early 2020 [8]. Conversely, to our knowledge, still no data on bronchiolitis epidemiology comprising the entire 2020–2021 epidemic season have been published. The aim of the present study was to assess the number of infants hospitalized in our pediatric emergency department (PED) with diagnosis of bronchiolitis during the ongoing pandemic, outlining any differences with previous years.

## 2. Materials and Methods

We retrospectively reviewed the medical records of children hospitalized with a diagnosis of bronchiolitis in the PED of the S. Orsola University Hospital (Bologna, Italy) between 1 October and 30 April of the epidemic seasons of 2017–2018, 2018–2019, 2019–2020 and 2020–2021. The Pediatric Department of S. Orsola Hospital is the largest Tertiary Pediatric referral Center in the E-R region, with approximately 24,000 admissions per year and serving a metropolitan area of more than 1 million people. Patient eligibility was defined through a hospital database search for diagnosis coded by the physicians at discharge according to the ICD (9th revision) (diagnosis codes 466.11 and 466.19). Those with bronchiolitis, with or without RSV infection, were included. Only infants aged <12 months at hospitalization were enrolled in order to limit potential overlaps with other respiratory conditions. Hospitalization criteria were defined according to the most recently updated Italian Inter-Society Consensus for bronchiolitis [9]. 

Oxygen therapy (either standard or high-flow) administration data were obtained. Respiratory syncytial virus (RSV) infection was routinely assessed at presentation in those suspected for bronchiolitis through a rapid antigenic test performed on swab specimens and subsequently confirmed by real-time PCR (rtPCR) by pediatricians and nurses with specific training in pediatrics. 

The proportion of bronchiolitis cases hospitalized during the March–April period within the corresponding season’s total was calculated and compared through Chi-square tests between the epidemic seasons of 2017–2018, 2018–2019 and 2019–2020. RSV infections and oxygen therapy administration rates for March-April 2017–2018, 2018–2019 and 2019–2020 were assessed and compared among seasons with Chi-square tests. Post-hoc season-to-season comparisons by Chi-square tests were subsequently performed where appropriate.

The results are expressed as percentages or the absolute number of cases as appropriate; *p*-values < 0.05 were considered statistically significant. Data analysis was performed with SPSS (IBM SPSS Statistics for Mac, Version 26.0. Armonk, NY, USA: IBM Corp). Graphs were produced with GraphPad (GraphPad Prism for Mac, Version 8.4.0). 

## 3. Results

**Figure 1:**
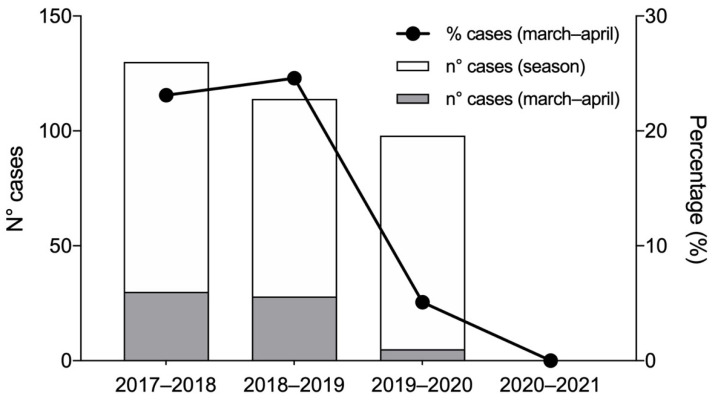
Severe drop in hospitalizations for bronchiolitis during March–April 2020 (5.1%, 5/98) as compared with 2019 (24.6%; 28/114) and 2018 (23.1%; 30/130), *p* < 0.001. No patients with bronchiolitis were hospitalized during the entire 2020–2021 (0) epidemic season.

A total of 342 children with diagnosis of bronchiolitis were included in our observation periods, specifically, 130, 114 and 98 for 2017–2018, 2018–2019 and 2019–2020, respectively. No infants with a diagnosis of bronchiolitis were hospitalized during the epidemic season of 2020–2021. Compared with the hospitalizations from October to April, patients hospitalized only in the March–April period were 30 (23.1%), 28 (24.6%) and 5 (5.1%) for 2017–2018, 2018–2019 and 2019–2020, respectively. A significant difference (*p* < 0.001) was reported when comparing the March–April hospitalization proportion of the three seasons. Post-hoc tests among the seasons showed a significant difference between March–April 2020 and the same period in 2018 (*p* < 0.001) and 2019 (*p* < 0.001). The data are presented in Figure 1.

The rate of infants who tested positive for RSV was 94/130 (72.3%) in 2017–2018, 75/114 (65.8%) in 2018–2019 and 72/98 (73.4%) in 2019–2020. The RSV infection rate among those hospitalized with bronchiolitis during March–April for the seasons of 2017–2018, 2018–2019 and 2019–2020 was 63.3% (19/30), 57.1% (16/28) and 60% (3/5), respectively (*p* = 0.89).

The rate of infants who underwent oxygen therapy was 56/130 (43.1%) in 2017–2018, 42/114 (36.8%) in 2018–2019 and 41/98 (41.8%) in 2019–2020. The oxygen therapy administration rate in infants hospitalized with bronchiolitis during March–April in 2017–2018, 2018–2019 and 2019–2020 was 33.3% (10/30), 57.1% (16/28) and 60% (3/5), respectively (*p* = 0.17). PICU admissions were 2.3% (3/130), 0% (0/114) and 1% (1/98) for the seasons of 2017–2018, 2018–2019 and 2020–2021, respectively. Moreover, no deaths for bronchiolitis before reaching hospital care or in children directly admitted to the pediatric intensive care unit occurred in the study period.

## 4. Discussion

Our data show a relevant reduction in hospitalizations for bronchiolitis after the first SARS-CoV-2 lockdown (March–April 2020), as compared with previous seasons (2017–2018 and 2018–2019), concordant with literature data worldwide. These findings were further confirmed by those from the epidemic season of 2020–2021, where no bronchiolitis cases were reported.

The drop in bronchiolitis observed in our center during March–April 2020 seems consistent with the closure of schools, kindergartens and daycares imposed by the Italian government as of 24 February to react to the SARS-CoV-2 outbreak. The literature data support the effectiveness of similar restrictions that had already been deployed on a smaller scale during other pandemics such as the H1N1 outbreak (2009), when school lockdowns in Japan led to an estimated decrease in the number of students infected at peak by over 24% [10]. As our population comprised infants and thus, by definition, they not attending such facilities, the measures imposed are more likely to have affected the bronchiolitis prevalence in an indirect rather than a direct way. Indeed, there is strong evidence supporting the key role of having older siblings attending school as one of the most important risk factors for the development of infectious diseases, including bronchiolitis [1,11]. Moreover, bronchiolitis is known to be caused mainly by RSV, the epidemiology of which is known to vary through different epidemic seasons. In order to address this possible bias, we included data from three epidemic seasons in our paper confirming a variability in RSV prevalence in our cohort (65.8–73.4%), coherent with the literature [1,12]. Even if, in our center, RSV remained the most represented pathogen in those with bronchiolitis during the first phase of the SARS-CoV-2 pandemic (March–April 2020), these data have to be read with regard to the severe overall reduction in bronchiolitis, which suggested a severe drop in RSV circulation.

The reduction in bronchiolitis hospitalizations observed during March–April 2020 may still not be fully explained only by the effect of pandemic-driven restrictions on infectious diffusion, as it has to be interpreted in the context of a dramatic reduction in PED attendance. It has been well documented how the SARS-CoV-2 pandemic has enhanced the perceived risk of gathering for hospital care, leading to an important decrease in the number of people seeking medical help. This reduction, similar to that described during previous outbreaks, such as SARS in 2003 [13], is supported by data for the ongoing pandemic, depicting an even more severe drop in PED visits (73% to 88%) during March 2020 (compared with the same period in 2019 and 2018), with an increased risk of more severe presentations [4,14]. In our population, this psychological factor seems to be less influential, since hospitalized patients with a defined diagnosis of bronchiolitis are, by definition, in need of in-hospital care now as before. Nevertheless, the proportion of patients on oxygen therapy was substantially similar during March–April 2020 and the same period in 2019 and 2018, ruling out any hypothetical selection for severe cases seeking hospital assistance possibly driven by fear of the hospital environment. 

Furthermore, our data from March 2020 appear to be in line with those from other European countries. A time series analysis by Angoulvant et al. in France reported a significant decrease in airborne-disseminated infectious diseases, including bronchiolitis (−63.5%), during the COVID19 outbreak [6]. Data from Belgium strengthen this evidence, outlining an early ending of the 2019–2020 bronchiolitis season, together with a nearly absent burden of disease in the first months of the epidemic season of 2020–2021 [15]. Our data point in the same direction, given that no bronchiolitis cases were reported in our center during the entire 2020–2021 season.

In order to better understand the significance of our results, note that the general lockdown with stay-at-home orders was maintained in Italy from 9 March 2020 until 4 May 2020 and again during autumn and winter of 2020–2021, this time with different restrictions, depending on the geographical area considered. Schools remained closed from 24 February 2020 until the end of the school year, and again between 1 March 2021 and 6 April 2021. However there has been wide variability, depending on the kind of school considered, with kindergartens and primary schools open for the most of the pandemic, while high schools implemented full distance learning or mixed distanced/in presence teaching [16]. As a consequence, we could speculate that since general lockdowns and school proscription for all age groups were maintained in Italy only for limited periods, our findings suggest a pivotal role for the milder NPIs such as the compulsory use of face masks and hand hygiene deployed for the whole duration of the pandemic. Evidently, any litmus test for these assumptions will become possible only once adherence to those measures fades. 

Several papers have raised some concerns on the possible occurrence of a so-called RSV “late peak” targeting a population of largely RSV-naïve infants, a hypothesis which seems to be supported by first-hand data from an Australian cohort described by Foley et al. [17]. To date, no reliable data describing any RSV late peak in Italy have been published. Geographic and socio-demographic differences, together with a great variability in the kind and timing of the public health interventions deployed, make a country to country comparison difficult, if not impossible. We have thus considered it appropriate to partially address this issue by enlarging our period of observation to include data from April in the2018, 2019, 2020 and 2021 seasons.

In conclusion, we report a significant drop in bronchiolitis hospitalizations in our center during the first SARS-CoV-2 lockdown (March–April 2020) and throughout the following epidemic season in 2020–2021. We could speculate that this phenomenon is to be ascribed mainly to the lower circulation of infectious agents, first of all RSV, secondary to the adoption of simple prophylactic measures such as hand hygiene and face masks rather than to lockdowns or school closures. Further evidence is needed to confirm and better quantify this trend, as careful and systematic adherence to these basic and cost-effective NPIs could play an even greater than thought role in controlling bronchiolitis epidemics in the years to come.

## Data Availability

The data presented in this study are available on request from the corresponding author.
